# Macrophages: Key Cellular Players in HIV Infection and Pathogenesis

**DOI:** 10.3390/v16020288

**Published:** 2024-02-13

**Authors:** Marie Woottum, Sen Yan, Sophie Sayettat, Séverine Grinberg, Dominique Cathelin, Nassima Bekaddour, Jean-Philippe Herbeuval, Serge Benichou

**Affiliations:** 1 Institut Cochin, Inserm U1016, CNRS UMR-8104, Université Paris Cité, 75014 Paris, France; marie.woottum@inserm.fr (M.W.); sen.yan@inserm.fr (S.Y.); sophie.sayettat@uk-koeln.de (S.S.); 2 CNRS UMR-8601, Université Paris Cité, 75006 Paris, France; severine.grinberg@u-paris.fr (S.G.); dcathelin.pro@gmail.com (D.C.); nassima_47@hotmail.fr (N.B.); jean-philippe.herbeuval@parisdescartes.fr (J.-P.H.)

**Keywords:** HIV-1, macrophages, virus replication, routes of infection, viral persistence, chronic inflammation

## Abstract

Although cells of the myeloid lineages, including tissue macrophages and conventional dendritic cells, were rapidly recognized, in addition to CD4+ T lymphocytes, as target cells of HIV-1, their specific roles in the pathophysiology of infection were initially largely neglected. However, numerous studies performed over the past decade, both in vitro in cell culture systems and in vivo in monkey and humanized mouse animal models, led to growing evidence that macrophages play important direct and indirect roles as HIV-1 target cells and in pathogenesis. It has been recently proposed that macrophages are likely involved in all stages of HIV-1 pathogenesis, including virus transmission and dissemination, but above all, in viral persistence through the establishment, together with latently infected CD4+ T cells, of virus reservoirs in many host tissues, the major obstacle to virus eradication in people living with HIV. Infected macrophages are indeed found, very often as multinucleated giant cells expressing viral antigens, in almost all lymphoid and non-lymphoid tissues of HIV-1-infected patients, where they can probably persist for long period of time. In addition, macrophages also likely participate, directly as HIV-1 targets or indirectly as key regulators of innate immunity and inflammation, in the chronic inflammation and associated clinical disorders observed in people living with HIV, even in patients receiving effective antiretroviral therapy. The main objective of this review is therefore to summarize the recent findings, and also to revisit older data, regarding the critical functions of tissue macrophages in the pathophysiology of HIV-1 infection, both as major HIV-1-infected target cells likely found in almost all tissues, as well as regulators of innate immunity and inflammation during the different stages of HIV-1 pathogenesis.

## 1. Introduction

Initially described, together with conventional dendritic cells (DCs) and monocytes, as cells of the mononuclear phagocyte system derived from a common myeloid progenitor, macrophages are found in almost all tissues of the body where they play several critical roles in tissue homeostasis, both in physiological and pathological conditions (for review, see [1]). While the role of non-differentiated blood monocytes as HIV-1 target cells was previously debated [2], it was recently reported that latent HIV-1 could be detected in monocytes in half of people living with HIV receiving effective combined antiretroviral therapy (cART) [3]. In contrast, terminally differentiated DCs and tissue macrophages were rapidly recognized, in addition to CD4+ T lymphocytes, as productively infected target cells of HIV-1 (for reviews, see [4,5,6,7]). However, macrophages are largely phenotypically and functionally heterogenous depending on their tissue distribution and their origin. While tissue-resident macrophages are specialized macrophages derived from an embryonic origin and differentiate locally to exert specific functions in the lungs (as alveolar macrophages), central nervous system (CNS) (as microglial cells), bones (as multinucleated osteoclasts), liver (as Kupffer cells), placenta (as Hofbauer cells), or as histiocytes within other interstitial connective tissues, almost all tissues can be populated by infiltrating macrophages derived from blood monocytes, especially in inflammatory conditions, contributing to their phenotypic diversity [1].

Although myeloid cells expressing the CD4 receptor and chemokine coreceptors for virus entry, and particularly tissue macrophages, were rapidly identified as target cells of HIV-1, studies regarding the mechanisms of viral replication in macrophages and their role in the pathophysiology of HIV-1 infection have been largely neglected. This fact is probably related to several reasons, but one of the main reasons was related to the major focus of the initial HIV research field on CD4+ T cells which were considered the main target cells of HIV-1, due to the spectacular decline of CD4 cell counts observed in HIV-infected patients with AIDS. Thus, HIV-1 infection and AIDS was, for a long time, considered a disease of the CD4+ T cell lineage.

However, macrophages have now emerged, over the last decade, as important HIV-1 target cells, likely involved in all stages of infection pathogenesis, including virus transmission and dissemination, as well as in viral persistence through the early establishment, together with latently infected CD4+ T cells, of virus reservoirs in many host tissues (for recent reviews, see [4,5,6,7]). This establishment of virus reservoirs is the major obstacle to virus eradication in people living with HIV (for review, see [8]). Infected macrophages are indeed found in a wide range of both lymphoid and non-lymphoid tissues in HIV-1-infected patients, including the CNS, lymph nodes, spleen, lungs, and digestive and genitourinary tracts. Therefore, macrophages play two significant roles during the natural course of HIV-1 infection, first as primary HIV-1 target cells, and then as central players of innate but also adaptive immunity. Macrophages also likely participate, directly or indirectly, in the chronic inflammation observed in people living with HIV, including patients on effective cART [4,5,6,7].

The complexities extend beyond the immediate impact on viral replication, delving into broader considerations of how HIV-1 manipulates macrophages both as viral host cells and reservoirs. Macrophages, distributed across diverse tissues, emerge as pivotal actors in facilitating virus dissemination to anatomical sites less amenable to conventional treatments. Their extended lifespan and capacity to harbor viral particles present an enduring challenge to achieving complete viral eradication.

Therefore, the general goal of this review is to provide an overview of the specific roles of macrophages as key cellular players in HIV-1 infection and pathogenesis. We will primarily discuss the presence, roles, and consequences of infected macrophages in almost all tissues, and then about the specificities of virus replication and/or restriction in macrophages as HIV-1 target cells through different modes of infection. The last part of the review will be focused on the role of macrophages in the chronic immune activation and inflammation observed in people living with HIV-1, including patients on effective cART. 

## 2. Macrophages as Cellular Targets of HIV-1 In Vivo

**Figure 1:**
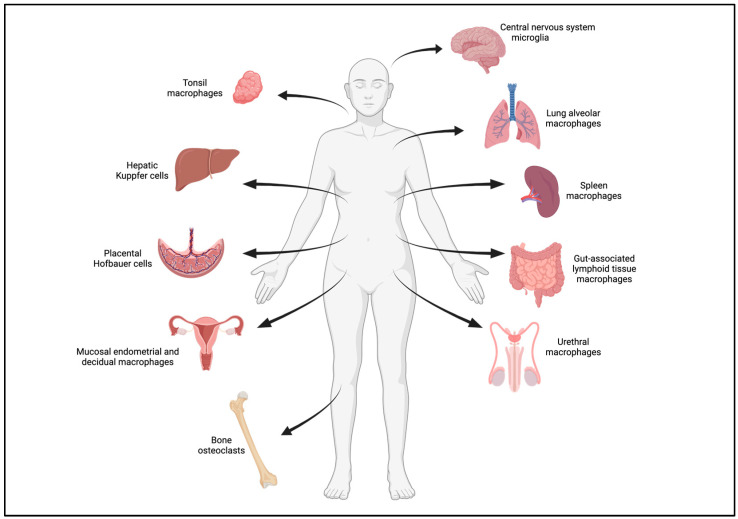
Anatomical localization of infected macrophages in vivo. The different tissues and organs where infected macrophages were detected in HIV-1-infected patients are indicated.

While it is generally accepted that HIV-1 primarily targets CD4+ T cells, cells of the myeloid lineage, including tissue macrophages, conventional dendritic cells (DCs), and bone osteoclasts (OCs), which all express the CD4 receptor as well as the CXCR4 and CCR5 coreceptors required for virus entry, are increasingly recognized as important productively HIV-1-infected target cells involved in the different steps of HIV-1 pathogenesis (for review, see [8]). During the course of HIV-1 infection, macrophages could participate, as target cells, first in virus transmission at the mucosal virus entry sites during sexual contact, and then in virus dissemination in both lymphoid and non-lymphoid tissues (see Figure 1). Infected macrophages can then efficiently transfer viruses to uninfected CD4+ T cells across a virological synapse [4,9]. However, additional investigations seeking to elucidate the specific role of macrophages in HIV-1 transmission, particularly in mucosal tissues, are needed to shed light on the early events that contribute to the establishment of an infection. Finally, infected macrophages are not susceptible to the virus-induced cytopathic effect, and can thus participate, together with latently infected CD4+ T cells, in the early establishment and persistence of virus reservoirs in numerous host tissues (for recent reviews, see [2,10]). As recently shown [3], these virus reservoirs in myeloid cells can be detected over several years in patients on cART. Remarkably, HIV-1-infected myeloid cells, including macrophages and DCs, are frequently detected in tissues as multinucleated giant cells (MGCs) [11,12,13,14,15,16,17,18,19,20,21].

### 2.1. Infected Macrophages in Tissues of People Living with HIV-1

Due to the prolonged lifespan of HIV-1-infected macrophages in tissues, they certainly represent one of the major obstacles to virus eradication and contribute to the persistence of adverse effects and associated disorders even in people living with HIV treated with cART [3,4,22,23,24,25,26]. As largely exemplified in early studies performed on post-mortem material, it is generally admitted that CD4+ T cells do not directly contribute to infection of the CNS. Instead, parenchymal microglial cells, the resident macrophage cell lineage, as well as infected perivascular monocytes/macrophages migrating through the blood–brain barrier, are the main cells infected by HIV-1. The presence of infected microglial cells and macrophages was associated with the development of HIV-associated neurocognitive disorders (HANDs) (for review, see [27]), which can persist in infected patients even on effective cART [11,25,26,28]. In the CNS of HIV-infected patients, infected myeloid cells are mostly detected as MGCs, and some authors initially proposed that the detection of MGCs could serve as a hallmark of AIDS-associated neurologic disorders [19,29,30].

Lungs are other tissues where macrophages could represent the main target cells of HIV-1, as revealed by the prevalence of infected alveolar macrophages in bronchoalveolar lavages of infected individuals [31,32,33], suggesting that alveolar macrophages could act as local pulmonary virus reservoirs with selective impairment of their phagocytic function [32]. In agreement, previous studies showed a severe inhibition of the phagocytosis functions of HIV-1-infected macrophages [34,35]. Therefore, pulmonary diseases, including chronic obstructive pulmonary disease, asthma, pulmonary hypertension, and even lung cancer, continue to be a major cause of morbidity and mortality for people living with HIV despite cART [31]. As expected, coinfection with *Mycobacterium tuberculosis*, the pathogen that causes tuberculosis, is also a risk factor in HIV-1-infected individuals, and several in vitro studies showed that the tuberculosis environment favors HIV-1 replication in macrophages [36,37,38,39].

In the liver, early studies reported that the resident Kupffer macrophages were infected by HIV-1, as evidenced by the detection of viral RNA, DNA, and antigens, in Kupffer cells from AIDS patients [40,41]. Since more recent studies have shown that purified Kupffer cells are highly permissive to HIV-1 infection in vitro, some authors propose that these resident macrophages could play important role in driving hepatic inflammation and fibrosis in infected patients (for review, see [42]). Similarly, HIV-1 infection of the placental Hofbauer macrophages was recognized early as they are central mediators of vertical mother-to-child HIV-1 transmission during pregnancy (for reviews, see [43,44,45,46]). Furthermore, it was recently reported that Hofbauer cells isolated from early- and mid-gestation are rather resistant to HIV-1 replication in vitro, compared to the higher susceptibility of term-gestation Hofbauer cells [47]. These observations likely show that mother-to-child HIV-1 transmission mainly occurs during the third trimester of gestation and delivery [43]. It was also reported that the local cytokine and hormonal environment could orchestrate the differentiation of mucosal endometrial and decidual macrophages to substantially modulate HIV-1 replication [45,48].

Intriguingly, it was suggested that gut macrophages are resistant to HIV-1 infection (for reviews, see [2,49]), even though it was reported that duodenal macrophages from gut biopsies of ART-suppressed people living with HIV can express viral antigens [50]. In vitro, it has been shown that intestinal macrophages are less permissive than cervicovaginal macrophages to HIV-1 infection and replication [51]. Interestingly, mucosal macrophage HIV-1 reservoirs have been recently described in urethral tissues from cART-suppressed patients [22,52]. These macrophages display the newly described specific CXCL4-induced M4 inflammatory phenotype expressing the calcium-binding protein A8 (S100A8) [53,54] and containing reactivable replication-competent HIV-1 particles [52]. The question remains open whether these urethral macrophage virus reservoirs are locally formed early after sexual transmission or later during HIV-1 infection progression. Finally, HIV-1 spreading in bone OCs [55,56,57,58], myeloid multinucleated cells involved in bone tissue homeostasis, could be responsible for the bone loss and disorders observed in infected patients, even when they are treated with efficient cART (for recent reviews, see [59,60]).

### 2.2. HIV-1 Infection of Tissue Macrophages and Polarization

In addition, macrophage susceptibility to HIV-1 infection and replication could also greatly vary in vivo depending on the wide range of macrophage activation states related to their tissue distribution and origin, and whether it is local or systemic inflammation, and thus, the cytokine environment during the distinct stages of HIV-1 infection and disease progression (for reviews, see [61,62,63]). In response to the local cytokine tissue environment, macrophages exhibit phenotypic and functional heterogeneity [64], and could differentially switch from a dominant pro-inflammatory (M1) program during the acute phase of HIV-1 infection to an anti-inflammatory (M2) program in the later stages [61]. Surprisingly, in vitro experiments performed using distinct cytokines to mimic M1 and M2 polarization showed that both M1- and M2-like MDMs are less susceptible to cell-free virus infection than unpolarized MDMs, suggesting an induction of programs to restrict virus replication in polarized macrophages [65,66]. Moreover, the activation state of macrophages varies during disease progression and is instrumental in modulating the efficacy of cell-free viral infection [62]. For example, in the endometrium of non-pregnant women and in the placental decidua, resident macrophages have a mixed M1/M2 phenotype [45]. When maintained in culture, these cells switch to a more M2 phenotype and become more permissive to cell-free viral infection [48]. However, in vitro polarization to M1 or M2 macrophages represent the extremes of a presumable continuum of polarization in vivo in distinct tissues. As mentioned above, mucosal HIV-1 reservoirs have been recently described in macrophages with the M4 inflammatory phenotype [52]. Together, these few studies reflect the complexity of the impact of macrophage differentiation and polarization in HIV-1 infection, and the need for further investigations in vivo and ex vivo of this important point in tissue-resident infected macrophages.

### 2.3. Animal Models for Investigating Infected Macrophages In Vivo

Although the frequency of macrophage infection during the disease progression of HIV-1-infected individuals had been initially discussed [67], it is now largely accepted that infected macrophages are found in a large range of tissues in vivo [22,32,49,68,69,70,71,72]. Importantly, investigations in experimentally infected animal models have confirmed the presence of infected macrophages in many tissues, as well as their critical role in pathogenesis. In agreement, immunohistochemical analyses performed in macaques infected with simian immunodeficiency viruses (SIVs) revealed the presence of infected tissue-resident and monocyte-derived macrophages in the lymph nodes, spleen, CNS, genital and gastrointestinal tracts, lungs, liver, and kidneys early after infection or under conditions of CD4+ T cell depletion, similar to what was observed at late stages of AIDS in HIV-1-infected patients [4,17,23,49,73,74,75,76,77,78]. Finally, the key role of macrophages in HIV-1 pathogenesis has been also demonstrated in HIV-1-infected humanized mice expressing only myeloid cells, where macrophages were sufficient to sustain productive infection in vivo, independent of CD4+ T cells [79]. As expected, HIV-1 infected macrophages were identified in different tissues, including the lungs, liver, spleen, and of course brain of these infected humanized mice. In addition, it was confirmed in this animal model that infected macrophages participate in the formation and maintenance of tissue virus reservoirs even in mice treated with cART [80,81,82,83]. To summarize, it is evident that these experimentally infected animal models certainly represent the best models, mainly for future in vivo investigations, but also for both ex vivo and in vitro studies, to analyze the specific and complex roles of macrophages in HIV-1 pathogenesis.

## 3. Cellular and Molecular HIV-1 Replication in Macrophages

As summarized above, macrophages are now emerging as crucial HIV-1 target cells in vivo [4]. However, the cellular and molecular mechanisms leading to productive HIV-1 infection and replication in these non-cycling terminally differentiated cells are still incompletely understood, and may substantially differ from those that have been extensively investigated in CD4+ T cells during the distinct early and late steps of the virus life cycle [9,84]. In vitro, cell culture experimental systems, mostly using macrophages derived from the differentiation of purified blood monocytes (MDMs) or the human promonocytic CD4+ THP-1 cell-line, definitively showed that these myeloid cells are difficult to infect with HIV-1 and are less susceptible to cell-free virus infection compared to activated primary CD4+ T cells [85,86,87]. Moreover, a few studies using primary macrophages isolated from tissues, such as bronchoalveolar, tonsillar, synovial, or placental macrophages, showed that tissue macrophages were often more refractory than MDMs to HIV-1 infection in vitro, indicating that human MDMs cannot recapitulate the characteristics and complexity of tissue-resident macrophages and their environment [4,44]. Although tissue macrophages are a biological material that is difficult to access, further investigations of HIV-1 infection of these cells is needed to try to decipher their characteristics and differences. This poor virus replication in macrophages was mainly associated with the high expression of host cell restriction factors, such as SAMHD1 (SAM and HD domain-containing protein 1), as part of the host innate immune intracellular response. However, the first block to efficient macrophage infection is related to virus entry and the specific cellular tropism of the different viral variants found in HIV-1-infected individuals. The aim of this paragraph is to highlight the differences and specificities of the different steps in and restrictions on the HIV-1 life cycle in macrophages, compared to the infection of CD4+ T cells.

### 3.1. Macrophage Tropism of HIV-1 Strains

The first and main block to efficient productive infection of macrophages is directly related to the virus entry and specific cellular tropism of the different viral variants isolated from HIV-1-infected individuals, at least in vitro in cell culture systems using MDMs or tissue macrophages [88,89,90]. The large majority of the HIV-1 strains used for in vitro analyses, usually isolated from the peripheral blood of infected patients, are mostly unable to infect macrophages in the cell-free in vitro infection assays used to characterize the cellular tropism of HIV-1 strains [91,92,93]. The current in vivo model is that HIV-1 strains that are able to infect macrophages emerge in tissues with a CD4+ T cell paucity such as the CNS, or at the late stages of disease progression when the CD4+ T cell count declines in untreated infected patients [73,94].

Due to its high mutability, HIV-1 isolates indeed exhibit high diversity in the viral gp120/gp41 envelope glycoproteins present at the surface of the virus particles, which are required for virus entry into lymphoid and myeloid target cells. The gp120 surface glycoprotein initially binds to CD4 and then to the CCR5 or CXCR4 chemokine receptor, leading to conformational changes in the gp41 transmembrane glycoprotein that finally mediates fusion of the viral envelope with the target cell membrane. The preferential usage of the CXCR4 and CCR5 coreceptors led to the definition of the viral tropism (X4-, R5-, and dual (X4/R5)-tropic viruses which use CXCR4, CCR5, or both chemokine receptors for virus entry, respectively) [95].

**Figure 2:**
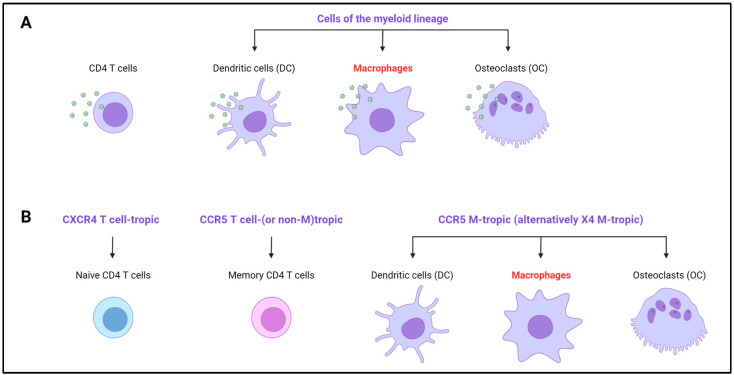
Cellular and viral tropisms of HIV-1 strains. (**A**) Cellular lymphoid and myeloid target cells of HIV-1. (**B**) Viral (co-receptor usage) and cellular tropisms of HIV-1 strains defined by in vitro infection assay.

More importantly for the scope of this review, the viral envelope also determines the cellular tropism of HIV-1 strains, which is defined as the target cell type (i.e., myeloid cells, including macrophages, and lymphoid CD4+ T cells) in which a specific viral strain can enter and then complete a full productive infectious replication cycle. According to this cellular tropism, the different HIV-1 isolates usually used for in vitro cell-culture experiments were classified into three different types: CXCR4-using T cell-tropic; CCR5-using T cell-tropic; and macrophage (M)-tropic strains, which mostly use CCR5, even if some X4-using and X4/R5-dual M-tropic virus strains have been reported (Figure 2) [91]. The first two categories are thought to represent the majority of viruses and are often also called non-macrophage (non-M)-tropic viruses. Importantly, the M-tropic viruses are able to enter and replicate in macrophages and dendritic cells but also in primary CD4+ T cells, whereas T cell-tropic viruses fail to enter into macrophages in vitro in cell-free infection assays, and can thus infect and replicate in CD4+ T cells only. The reasons why most HIV-1 isolates are non-M-tropic and fail to enter into macrophages is not really fully understood, but this feature has been generally explained by a lower efficiency of interactions between the viral envelope and the virus entry receptors on macrophages than on CD4+ T cells [91]. In agreement, the viral envelopes of many M-tropic HIV-1 isolates, mostly isolated from the CNS [70], often exhibit higher affinities for the CD4 receptor expressed at the cell surface of macrophages than the envelope of T cell-tropic viruses [70,96,97,98,99,100,101]. Therefore, T cell-tropic viruses require a higher density of CD4, as expressed on CD4+ T cells, for efficient entry [91,96,102]. In addition, enhanced interactions of M-tropic virus envelopes with the CCR5 or CXCR4 coreceptors have been also reported [103,104,105,106]. Together, these results likely explain why M-tropic viruses often show increased resistance to inhibitors of virus entry, such as neutralizing anti-envelope antibodies and antagonist drugs targeting the CCR5 and CXCR4 coreceptors [100,104,107].

Thus, the current view of the literature is that HIV-1 isolates fully equipped to infect macrophages and other myeloid cells are uncommon [91], especially during the early and asymptomatic stages of infection. In contrast, genuine M-tropic viruses are thought to emerge in infected patients through adaptation to body niches where macrophages or related myeloid cells are present in a context of CD4+ T cell paucity, such as in the CNS, and at the late stage of infection when the CD4 T cell count declines. However, this general view remains paradoxical and could be challenged due the early presence and detection of HIV-1-infected macrophages in a wide range of tissues (see Figure 1) [22,68,70,108] and their capacity to promote systemic dissemination of the virus, as evidenced in SIV-infected monkeys and HIV-1-infected humanized mice [49,75,79,80,82]. We could thus speculate that one possible explanation for this apparent paradox is that the in vitro infection assays and cell culture systems used to classify the HIV-1 cellular M-tropism of the viral isolates do not fully recapitulate the different modes of macrophage infection in vivo [85].

### 3.2. Specific Modes of Cell-to-Cell Infection of Macrophages

**Figure 3:**
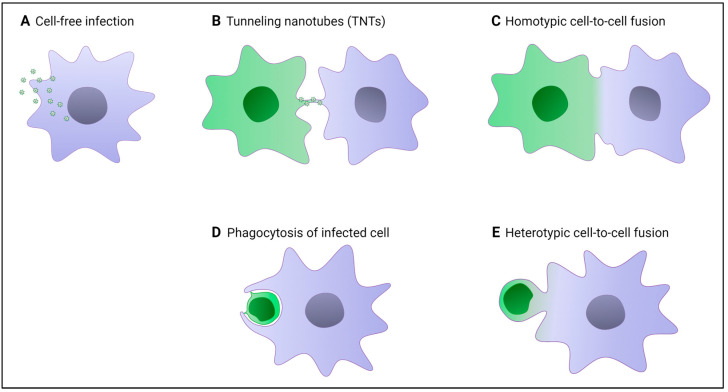
Intercellular structures and processes involved in cell-to-cell transfer of HIV-1 to myeloid cells. Different pathways for HIV-1 cell-to-cell transfer between virus-infected donor cells (in green) and target myeloid cells (in purple) have been described, in addition to cell-free virus infection (**A**), through the formation of tunneling nanotubes (TNTs) (**B**), homotypic cell–cell fusion (**C**), phagocytosis of infected T cells (**D**), and heterotypic cell–cell fusion with virus-donor infected T cells (**E**).

In addition to infection by cell-free virus particles (see Figure 3A), it was proposed that cell-to-cell transfer of HIV-1 after tight contact between infected virus-donor cells and recipient target cells likely represents the dominant mode of virus dissemination in vivo and may allow for the productive infection of both lymphoid and myeloid target cells [9]. Importantly, this mode of virus dissemination may also enable the virus to escape elimination by the immune system, including through neutralizing antibodies, as well as antiretroviral drugs, and to bypass the limitation of viral replication by the host cell SAMHD1 restriction factor [58,109,110,111,112]. At least in vitro, this cell-to-cell mode of virus transfer is very efficient for virus dissemination into target cells, such as macrophages and dendritic cells, which are poorly susceptible to cell-free virus infection [86,89]. While HIV-1 cell-to-cell transfer between CD4+ T cells or from infected macrophages or dendritic cells to CD4+ T target cells [9] have been largely explored, only a few studies investigating the mechanisms involved in cell-to-cell infection of macrophages have been reported so far [85]. These reports demonstrated that myeloid cells can be highly productively infected in vitro through virus cell-to-cell transfer between infected and uninfected macrophages, or from infected virus-donor CD4+ T cells to macrophage targets (see Figure 3). In addition, macrophages and DCs are also able to capture HIV-1 particles independently of CD4, using lectin receptors expressed on myeloid cells such as Siglec-1/CD169, and to transmit viruses to bystander CD4+ T cells through a mechanism called *trans*-infection, without virus replication in the myeloid cells [113,114]. In this process of Siglec-1-mediated virus capture, virus particles accumulate in a virus-containing compartment (VCC) before direct transmission to CD4+ T cell targets for replication [115,116].

#### 3.2.1. Homotypic HIV-1 Cell-to-Cell Transfer between Macrophages

The formation of thin or thick tunneling nanotubes (TNTs) (for review, see [117]), protrusions that extend from the plasma membrane and enable cells to touch over long distances (up to 100 μm), was proposed as an efficient mechanism to transport HIV-1 components and even virus particles between macrophages [118,119,120,121] (Figure 3B). Interestingly, the formation of TNTs and virus cell-to-cell transfer and replication are facilitated in HIV-1-infected macrophages in the context of coinfection with *Mycobacterium tuberculosis* (Mtb), the pathogen causing tuberculosis, through the induction of Siglec-1 expression [36,37].

Additionally, the intrinsic propensity of macrophages to mediate cell–cell fusion to form multinucleated cells, both in physiological and pathological conditions (for review, see [122]), has also been proposed as a potential mechanism for homotypic HIV-1 cell-to-cell spreading between macrophages (Figure 3C). This process results in the formation of infected multinucleated giant cells (MGCs), as observed in vivo in tissues of HIV-1-infected patients and experimentally SIV-infected macaques, mainly in the CNS, but also in other lymphoid or nonlymphoid tissues [14,16,19,20,29,123,124,125]. In addition, cell–cell fusion between infected and non-infected macrophages is also likely favored by the stimulation of TNT formation observed in infected macrophages [37,120,126], since the two cellular processes are positively modulated by the viral Nef auxiliary protein [126,127]. Despite their large size, these HIV-1-infected multinucleated macrophages migrate faster than their infected mononucleated counterpart and may favor virus dissemination in tissues [126]. Therefore, it is now well documented and accepted that viruses from different viral families, including human pathogens such HIV-1 and SARS-CoV-2, can mediate cell–cell fusion and syncytia formation between infected cells and neighboring non-infected cells for in vivo virus spreading, which was evidenced in tissues such as the brain, lymphoid tissues, and lungs of infected patients [128,129,130].

#### 3.2.2. Heterotypic HIV-1 Cell-to-Cell Transfer from Infected CD4+ T Cells to Myeloid Target Cells

Two different modes of heterotypic HIV-1 cell-to-cell transfer from infected CD4+ T cells to macrophage targets have been described [131,132]. The first mode of cell-to-cell virus infection described was related to the main biological role of macrophages to detect and eliminate dying and infected cells through phagocytosis [1] (Figure 3D). These authors showed that HIV-1-infected CD4+ T cells, preferentially when apoptotic, can be selectively engulfed by macrophages [131]. Only CD4+ T cells infected by CCR5-using M-tropic strains and, to a lesser extent, CCR5-using T/F viruses, which poorly infect macrophages in cell-free infection assays [133,134], led to productive infection of the target macrophages [131]. By contrast, no productive infection of the macrophage targets was observed after engulfment of T cells infected with non-M-tropic CXCR4-using viruses, even though these infected T cells were efficiently engulfed by macrophages. Surprisingly, this mechanism of engulfment of infected T cells is independent of interactions between the viral envelope glycoproteins and the cellular CD4 and CCR5/CXCR4 coreceptors. However, the subsequent step leading to productive infection of the macrophage target relies on interactions between the viral envelope and the CD4 receptor and the CCR5 coreceptor, as it is inhibited by antibodies or drugs targeting Env and CD4, and to a lesser extent by antagonists of CCR5. Interestingly, a recent finding reported that the Vpu membrane-associated HIV-1 auxiliary protein, which facilitates viral dissemination, can enhance the susceptibility of macrophages to infection through the phagocytosis of infected CD4+ T cells [135]. By inducing the down-regulation of CD47 from the surface of infected CD4+ T cells, a ligand of the inhibitory “don’t eat me” receptor signal regulatory protein-alpha (SIRPα) expressed on myeloid cells, Vpu thus promotes the phagocytosis of infected T cells by macrophages, leading to productive infection of these target cells by CCR5-using M-tropic viruses and also by non-M T/F viruses [135].

Finally, the second and most studied mode of HIV-1 cell-to-cell infection of macrophages is related to the new mechanism we have documented that leads to rapid and massive virus transfer from infected CD4+ T cells to myeloid cells, including macrophages and also DCs and bone OCs, by a two-step viral envelope-dependent cell–cell fusion mechanism (Figure 3E) [57,58,89,90,132,136]. In the first step, infected T cells establish contact with myeloid cells, resulting in cell–cell fusion with these target cells. The newly formed Gag+ fused cells are then able to fuse with surrounding non-infected macrophages, DCs, or OCs, leading to the formation of MGCs containing at least one nucleus coming from the initial fusion of infected T cells, and able to survive for a long period of time in cell culture and to produce high levels of fully infectious viruses [58,90,132].

Interestingly, this two-step cell–cell fusion mechanism for massive virus cell-to-cell transfer between infected CD4+ T cells and myeloid cell targets bypasses the inhibition of HIV-1 replication related to the high expression of the SAMHD1 host cell restriction factor observed when these myeloid cells are infected with cell-free viruses [137,138,139]. In infected MGCs formed through cell–cell fusion between infected T cells and macrophages, SAMHD1 is expressed in its inactive phosphorylated form, explaining how MGCs can survive for a long time in vitro while producing large amounts of virus particles [58,140,141]. In addition, we recently obtained unpublished results showing that the expression of the Nef, Vif, Vpu, and Vpr viral auxiliary proteins, known to counteract SERINC (serine incorporator), APOBEC3 (apolipoprotein B mRNA editing enzyme polypeptide-like 3), BST-2 (bone marrow stromal antigen 2), and other restriction factors did not affect the cell-to-cell spreading to macrophages from infected CD4+ T cells, suggesting that this cell–cell fusion mode for HIV-1 infection of macrophages may also bypass these restrictions factors (SB and MW, unpublished results).

At least in vitro, we have recently shown that this process of cell–cell fusion is the main and most efficient mechanism for HIV-1 cell-to-cell transfer from living infected T cells to macrophage targets, surpassing other processes such as phagocytosis [90]. Moreover, this process of cell-to-cell virus spreading is efficient for the productive ex vivo infection of purified primary tissue macrophages from various origins by fusion with infected CD4+ T cells. As previously mentioned, infected macrophage-derived MGCs, and also DC-derived MGCs, are found in vivo in tissues of HIV-1-infected patients. Infected macrophages are indeed frequently detected as multinucleated cells in the CNS and other tissues of HIV-1-infected patients and SIV-infected monkeys [11,14,16,19,20,29,76,123,142,143,144,145]. Interestingly, it was recently shown that these MGCs are active sites of virus replication in neuroparenchyma tissues of infected macaques with SIV-induced encephalitis [145]. In addition, this heterotypic and two-step cell–cell fusion process described in vitro for virus transfer to myeloid cells also agrees with the in vivo observations showing that T cell markers are detected in infected myeloid cells found in lymphoid tissues of SIV-infected macaques [73,74,75].

Initially revealed using HIV-1 strains expressing CCR5-using M-tropic viral envelopes, we have recently showed that primary viral variants isolated from infected patients, using either CCR5 and/or CXCR4 and characterized by cell-free infection assays as non-M tropic, including some R5-tropic T/F viruses, can be efficiently transferred from infected T cells and then disseminate in macrophages through cell–cell fusion, resulting in the formation of productively infected MGCs [89]. Although the viral tropism of the virus strains, defined by the coreceptor usage, is generally maintained between cell-free and cell-to-cell infection processes, the initial block of virus entry for efficient replication in macrophages could be overcome in T cell-to-macrophage cell–cell fusion. These findings suggest that HIV-1 strains have a broader tropism for macrophages than initially presumed. In agreement, a recent report showed that non-M-tropic primary HIV-1 isolates can efficiently infect alveolar macrophages through contact with infected CD4+ T cells [33]. Altogether, these findings indicate that the cellular tropism of primary HIV-1 isolates needs to be revisited to reflect the capacity of both CCR5- and CXCR4-using non-M-tropic variants, including some T/F viruses, to spread efficiently in myeloid cells via cell-to-cell transfer from infected T cells by cell–cell fusion. Compared with cell-free infection, our results show an enhancement of interactions between both CCR5-using M- and non-M-tropic viral envelope glycoproteins and CD4 and CCR5 during T cell/macrophage contact. Enhanced envelope/receptor interactions may increase the capacity of HIV-1 envelopes, and particularly non-M-tropic envelopes, to trigger cell–cell fusion of infected T cells with macrophages. In addition, we have recently shown that cell–cell fusion of infected T cells and virus spreading in macrophages are favored when macrophages are differentiated from blood monocytes in anti-inflammatory (M2) conditions compared to pro-inflammatory macrophages (M1) [90]. This could be explained, at least in part, by the reduction in the CD4 cell surface expression on pro-inflammatory macrophages [65,90]. Alternatively, the differences in the transcriptional programs induced in anti- or pro-inflammatory conditions could also modulate the sequential events leading to the fusion of infected CD4+ T cells with macrophages.

These processes of HIV-1 cell-to-cell spreading to myeloid cell targets also suggest that infection of macrophages from infected T cells might play a role in HIV-1 transmission. As mentioned above, cell-free virus infection assays led to the conclusion that sexually transmitted R5 transmitted/founder (T/F) viruses have little or no ability to infect macrophages, suggesting that macrophages do not contribute to early virus dissemination after transmission [92,146]. However, HIV-1 establishes new infections at mucosal surfaces and then in neighboring draining lymph nodes, which are both heavily populated with CD4+ T cells and myeloid cells including macrophages. Several studies have also reported that macrophages can be infected at the mucosal sites of HIV-1 transmission [22,50,51,147]. Viral cell-to-cell spreading from infected T cells supports the view that, at the sites of mucosal transmission, resident macrophages (and/or DCs) may be efficiently infected by non-M-tropic T/F viruses through cell-to-cell transfer from infected T cells, which then contribute to virus dissemination [89,131,135,136]. While R5 viruses, including T/F viruses, predominate in the early, acute, and asymptomatic phases of infection, dual-tropic R5/X4 and X4 variants can then emerge at a later stage of infection in some patients, when the proportion of CCR5-positive, memory CD4+ T cells drops in the peripheral blood, but resting naïve CD4+ T cells expressing mainly CXCR4 are still maintained [91,148]. Since we showed that R5/X4 and X4 viruses are very efficiently cell-to-cell transferred to macrophages [89,136], infected naïve CD4+ T cells could then transfer CXCR4-using viruses to tissue macrophages, thereby contributing to dissemination at later stages of HIV-1 infection.

Together, these recent findings regarding the different mechanisms used for HIV-1 cell-to-cell spreading to macrophages (Figure 3) likely impact different stages of the pathophysiology of HIV-1 infection regarding (i) viral sexual transmission through genital and rectal mucosae, (ii) viral spreading to target cells and to lymphoid and non-lymphoid tissues, and (iii) establishment of viral tissue reservoirs.

### 3.3. HIV-1 Replication (and Restriction) in Macrophages

**Figure 4:**
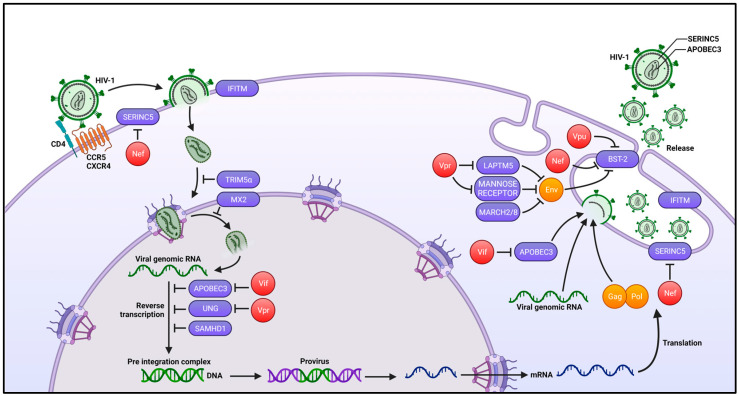
Schematic representation of the different early and late steps of the HIV-1 replication cycle in macrophages, with the potential host cell restriction factors shown in purple, while the counteracting viral auxiliary proteins are shown in red; structural viral proteins coded by the gag, pol, and env genes are in orange.

In contrast to the proliferative status of activated CD4+ T cells, which constitutes a favorable environment for efficient HIV-1 replication, it is evident that the non-dividing state of terminally differentiated macrophages should likely impact virus replication to prevent the completion of the distinct early and late steps of the virus life cycle. In addition to the first block related to the cellular tropism and modes of infection, the second main limitation responsible for the poor HIV-1 replication in macrophages, at least in vitro, was associated with the high expression of host cell restriction factors, which are mostly upregulated by interferons (IFNs) generated early in response to the viral infection as part of the innate immunity [149] (Figure 4). As mentioned above, the main and most studied cellular restriction factor is SAMHD1, but members of the SERINC (serine incorporator), IFITM (interferon-induced transmembrane), TRIM (tripartite motif), and APOBEC3 protein families, as well as Mx2/MxB, BST-2 (also called tetherin), and some other cellular proteins have also been described [149,150]. However, some of these restriction factors are counteracted by viral auxiliary proteins, including the HIV-1 Vif, Nef, Vpu, and Vpr proteins (Figure 4), as well as the Vpx protein expressed by HIV-2 and some SIV lineages but is absent from HIV-1. Deletion of these regulatory viral genes usually significantly impacts cell-free virus replication in primary target cells, such as macrophages, compared to wild-type viruses [149]. The main role of the HIV-1 auxiliary proteins, such as Nef, Vif, and Vpr, is to prevent the incorporation of host cell factors into progeny virus particles; these factors can act in the next cycle of infection to restrict the early steps of virus replication, including virus fusion at the cell surface, uncoating, reverse transcription, and then nuclear translocation and integration of the viral DNA. Only Vpu mainly acts at the late steps of the virus life cycle to promote the assembly, budding, and release of virus particles, even if some recent reports indicate that Vpr may also act on the late steps of replication to release virus particles with optimal infectivity (see below). Whereas the different steps of virus replication and restriction in lymphoid CD4+ T cells were widely investigated, there is a paucity of information regarding the role of viral auxiliary proteins and host cells restriction factors for virus replication in myeloid cells, such as macrophages. Here, we will focus below on the steps of the HIV-1 replication cycle involved in infection and/or restriction, specifically in myeloid cells (see Figure 4).

#### 3.3.1. Early Steps of the Virus Life Cycle

SAMHD1 is certainly the main cellular factor that limits efficient HIV-1 replication in macrophages, and is thus responsible for the poor susceptibility of this myeloid cell type, at least in vitro [151,152]. SAMHD1 is a triphosphohydrolase that is able to hydrolyze dNTP, leading to a reduction in the intracellular dNTP pool available for the reverse transcription process [139,141]. SAMHD1 activity is not counteracted in HIV-1, but the presence of the viral Vpx protein in HIV-2 and some SIVs leads to the degradation of SAMHD1 by the proteasome machinery allowing for the efficient replication of these lentiviruses in myeloid cells [138,139]. As mentioned above, the susceptibility to SAMHD1 restriction may also vary depending the mode of macrophage infection. When noncycling myeloid target cells, such as macrophages and DCs, are infected with cell-free viruses, SAMHD1 is expressed in its dephosphorylated active form and is able to restrict HIV-1 replication [153,154,155]. In contrast, we have shown that the cell–cell fusion process for cell-to-cell virus spreading from infected CD4+ T cells to myeloid cells resulted in the phosphorylation of SAMHD1, allowing for very efficient virus replication and spreading in macrophages and DCs [58].

Like SAMHD1, most restriction factors, including members of the SERINC, IFITM, TRIM, and APOBEC3 protein families as well as the Mx2/MxB protein, also inhibit entry or the post-entry early steps of the viral life cycle, before the integration of the viral DNA into the host genome [149,150]. Members of the IFITM (IFITM1, 2, and 3) and SERINC (SERINC3 and 5) protein families are known to restrict the entry of several enveloped viruses including HIV-1 by antagonizing fusion of the viral envelope with the cell membrane [156,157]. The mechanisms by which IFITM1, 2, and 3, and SERINC3 and 5 proteins impair virus infectivity are not fully defined, but these proteins modulate cell-free virus entry in target cells when they are incorporated into virus particles. Although SERINC proteins are known to be counteracted by the viral Nef protein through preventing their incorporation into the newly released virus particles in primary CD4+ T cells, nothing is known regarding the activity of SERINC3 or 5 proteins in the restriction of HIV-1 replication in macrophages. Interestingly, we recently confirmed previous published results indicating that the infectivity of Nef-deleted viruses was not significantly affected in macrophages as it is in CD4+ T cells [126], suggesting that SERINC proteins do not exert significant antiviral restriction activity in macrophage targets, even if it was recently reported that SERINC5 expression was upregulated in terminally differentiated macrophages [158].

After virus entry, uncoating of the incoming viral capsid may be restricted by TRIM family proteins, inducing premature disassembly and degradation of the viral capsid lattice (for review, see [159]). Because the most well-studied TRIM protein, TRIM5α, was historically considered to be unable to bind and restrict the HIV-1 capsid in human target cells, only a few studies have investigated the role of TRIM5α in the modulation of HIV-1 replication in human primary macrophages, although it was initially shown that TRIM proteins could modulate and block HIV-1 infection more strongly in non-dividing cells such as macrophages than in dividing cells [160]. Importantly, it has been recently shown that TRIM5α restriction of HIV-1 replication in infected cells, including macrophages, is counteracted by the protective interaction of the cyclophilin A protein with the incoming viral capsid [161]. Alternatively, other authors have suggested that TRIM5α was able to limit HIV-1 replication in human myeloid cells independently of its action on the viral capsid, through a mechanism related to a non-canonical role of the autophagy machinery [162]. More recently, it has been reported that another poorly studied member of the TRIM family, TRIM69, is able to restrict HIV-1, HIV-2, and SIV infection, specifically in interferon-stimulated myeloid cells through acting on the microtubule network [163]. Interestingly, TRIM69 is also able to restrict the replication of vesicular stomatitis, dengue, and SARS-CoV-2 viruses [164,165,166].

APOBEC3 proteins, mainly APOBEC3G and APOBEC3F, have been well-characterized as cellular factors that strongly restrict HIV-1 replication in primary cells, including both CD4+ T cells and macrophages (for review, see [167]). APOBEC3 proteins are cytidine deaminases that catalyze cytidine deamination into uracil in single-stranded DNA and thus interferes with the process of reverse transcription, resulting in a high frequency of G-to-A hypermutations in the proviral DNA. Since these enzymes are able to remove the amino group from cytosine bases, creating uracil residues in the viral DNA, it was suggested that the uracil DNA glycosylase (UNG2), an enzyme of the base excision DNA repair system that specifically removes the RNA base uracil from DNA, could then introduce a-basic sites in place of deoxyuridine, leading to the degradation of the neo-synthesized viral DNA [168,169]. The main counteracting function of the viral Vif protein is thus to target APOBEC3 proteins for proteasomal degradation in virus-producing cells, thus preventing their incorporation into newly formed virus particles [170,171,172,173]. Similarly, Vpr could induce proteasomal degradation of UNG2 to prevent its incorporation into virions [168,174]. However, other studies have suggested that the recruitment of UNG2 into virus particles, through direct interactions with Vpr, positively influences the accuracy of the reverse-transcription process and has a positive impact on HIV-1 infectivity and replication in macrophages [175,176,177]. As a component of the viral DNA-containing pre-integration complex, it was also suggested that Vpr may participate in the intracellular transport and nuclear translocation of the viral DNA in infected macrophages, but this Vpr function is still poorly characterized [178,179].

Finally, the Mx2 protein, also known as MxB, is a dynamin-like GTPase that also restricts viral replication by inhibiting the nuclear translocation of the viral DNA and pre-integration complex (for review, see [180]), but the specific restriction activity of Mx2 during HIV-1 replication in macrophages was not really investigated. In addition, it would be interesting to investigate the function of Mx2 and other restriction factors, such as TRIM, APOBEC3, UNG2, and SAMHD1, in the distinct post-entry steps of the viral life cycle, in relation with the new concept of late nuclear capsid uncoating after virus entry, which was evidenced by several groups, especially in HIV-1-infected macrophages [181,182,183,184], but also in CD4+ T cells [185]. While low amounts of intact capsids were detected in the nucleus compartment of HIV-1-infected CD4+ T cells, stronger nuclear capsid signals were detected in infected macrophages, indicating that capsid uncoating and completion of the reverse transcription process specifically occurs in the nucleus rather than in the cytoplasm during the infection of these myeloid target cells (for reviews, see [186,187]).

#### 3.3.2. Late Steps of the Virus Life Cycle

The BST-2/tetherin protein is the best-characterized restriction factor acting in the late steps of the viral life cycle by tethering virus particles at the cell surface of infected cells (for review, see [188]) (see Figure 4), leading to a limitation of virus budding and release during HIV-1 replication both in CD4+ T cells and in macrophages [189,190,191]. In infected macrophages, the assembly of the viral components and then the budding of de novo formed virus particles specifically take place in a specialized intracellular membrane compartment called the VCC, for virus-containing compartment (for reviews, see [6,192]). The VCC was described as a protected compartment, distinct from endosomes or multivesicular bodies, in continuity with the plasma membrane and connected to the extracellular space by thin channels. While the interplay between the cellular functions of BST-2 and the formation of the VCC in infected macrophages is still being discussed (for reviews, see [6,188]), the viral Vpu protein counteracts BST-2's tethering activity by promoting cell-surface downregulation of BST-2 through sorting to lysosomal compartments for degradation [193,194,195]. In addition, it is well documented that Vpu also interacts and retains neo-synthesized CD4 molecules in the endoplasmic reticulum for proteasomal degradation, leading to optimal cell surface expression of the viral envelope glycoproteins, and thus contributes to the optimal infectivity of the progeny virus particles [188]. Similarly, it was reported that Vpr, together with Nef, also participates in HIV-1-infected macrophages in the proper processing and expression of the viral envelope for the optimal release and spreading of fully infectious virus particles [196,197]. While some authors have reported that this Vpr activity was due to an indirect modulating transcriptional effect on the expression of the IFITM3 protein [198], other authors have suggested that it was related to the modulation of the mannose receptor, a C-type lectin expressed on the cell surface of almost all tissue macrophages, which could also act as an antiviral restriction factor through direct binding to the high-mannose glycan structures of the viral envelope glycoproteins [199,200,201]. More recently, a third factor counteracted by Vpr, called LAPTM5 (for lysosomal-associated transmembrane protein 5), that specifically restricts HIV-1 replication in both macrophages and dendritic cells, has been characterized [202,203]. This lysosomal protein might be involved in the inhibition of the late step of the viral envelope trafficking for the optimal infectivity of the released virus particles. By inducing the degradation of LAPTM5, Vpr may prevent the lysosomal degradation of the viral envelope to promote the optimal infectivity of the progeny virus particles released from macrophages. Similarly, E3 ubiquitin ligases of the membrane-associated RING-CH (MARCH) family, including members that are highly expressed in myeloid cells, such as MARCH1, 2, and 8, have been reported to restrict the viral replication and infectivity of HIV-1 through the downregulation of the cell surface expression of the viral envelope glycoproteins on macrophages [204,205,206].

To summarize, with the exception of SAMHD1 which was extensively analyzed in myeloid target cells, the specific role of most of these host cell restriction factors in the modulation of virus replication in macrophages has been often poorly or not analyzed. Further investigations of their restriction functions, as well as the modulating role of the counteracting viral auxiliary proteins, which also depend on the different modes of infection (cell-free or cell-to-cell), are thus needed for a better understanding of their respective roles during the viral life cycle, specifically during viral replication in macrophages.

## 4. Macrophages and HIV-Related Immune Responses and Inflammation

### 4.1. Macrophages and HIV-1 during Early Acute Phase of Infection

During HIV-1 sexual transmission, macrophages, along with CD4+ T cells, conventional dendritic cells (DCs), and plasmacytoid dendritic cells, encounter the virus in the genital and rectal mucosae. They play crucial roles in both the innate and adaptive immune responses to HIV-1 during the early stages of infection [207,208]. Due to their primary function of phagocytosis and clearance of viral particles and infected cells together, macrophages play two significant roles during the natural course of HIV-1 infection.

**Figure 5:**
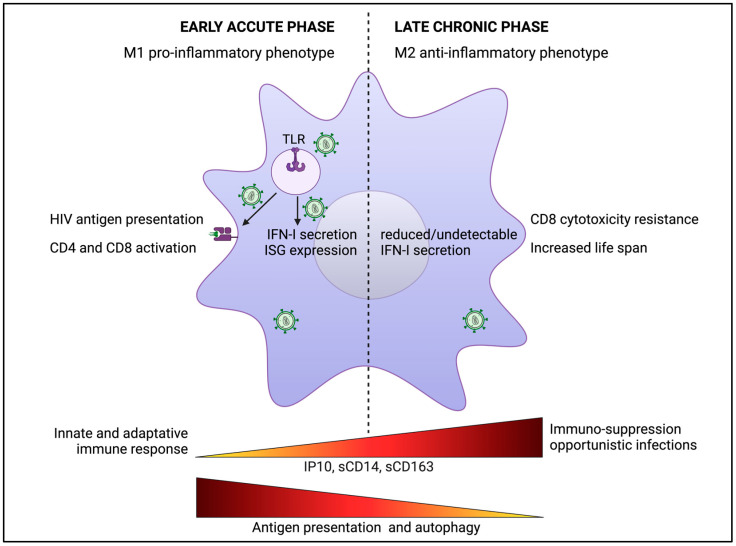
Macrophage activation state during HIV-1 infection. HIV-1 recognition by TLR7 induces macrophage activation and their transition from an inactivated to an M1 pro-inflammatory phenotype. The secretion of pro-inflammatory cytokines and chemokines induces innate and adaptative immune responses during the early phase of infection through massive IFN-I secretion and HIV-1 antigen presentation to T cells. The depletion of infected T cells and other target cells leads to an IL-4- and IL-13-rich environment responsible for the switch from the M1 to M2 anti-inflammatory phenotype of infected macrophages with a long life span, undetectable IFN production, and CD8 cytotoxic resistance. These anti-inflammatory M2 macrophages are permissive to new microbial agents and favor opportunistic infections despite the presence of soluble monocyte/macrophage activation markers.

On one hand, their ability to act as antigen-presenting cells (APC) allows them to present viral antigens to CD4+ T cells via the major histocompatibility complex (MHC) class II pathway, and to trigger CD8+ cytotoxic T cells (CTL) by cross-presentation [209,210,211]. Moreover, macrophages exhibit an important autophagy activity, allowing them to control the HIV-1 infection and enhance viral antigen presentation [212,213,214]. Therefore, the interaction between macrophages and T cells is of paramount importance in HIV-1 transmission and the immune response [215,216,217] (Figure 5).

On the other hand, during the early phase of infection following sexual transmission when viruses are not yet detectable in the blood [218], the viruses encounter immune cells and may potentially replicate in the dendritic cells and macrophages present in genital and rectal submucosal tissues. In this context, the Toll-like receptor (TLR) pathways also play essential roles in the innate immune responses related to macrophage functions (Figure 5) [219,220,221,222]. The activation of macrophages is primarily triggered by the TLR-mediated recognition of HIV-1 RNA and its pathogen-associated molecular patterns (PAMPs) released by infected cells. Therefore, this recognition prompts macrophages to adopt an M1 pro-inflammatory phenotype associated with the secretion of Th1 cytokines and chemokines (IFN-γ, IL-2, IL-12, and CCL3, CCL4, CCL5, respectively) and proinflammatory cytokines (TNF-α, IL-1β, IL-6, and IL-18) [223].

In addition, cytosolic macrophage DNA sensors may sense the viral HIV-1 DNA products generated during the reverse transcription (RT) process [224]. These viral DNA products are recognized by the cytosolic cyclic GMP–AMP synthase (cGAS), leading to the generation of circular GMP–AMP (cGAMP) dinucleotides. Then, cGAMP activates receptor stimulator of interferon genes (STING), initiating type I interferon (IFN-I) responses in macrophages through the TANK-binding kinase 1 (TBK1)/Interferon Regulatory Factor 3 (IRF3) axis [225]. TBK1 is a central serine/threonine protein kinase that mediates the phosphorylation and nuclear translocation of IRF3, contributing to the induction of type I IFNs in the innate antiviral response. Alternatively, other studies have shown that HIV RT DNA intermediates trigger IFN-I production in macrophages in an Interferon Gamma-Inducible Protein 16 (IFI16)/STING-dependent manner [226,227]. All these processes should result in the inhibition of viral replication and the activation of the adaptive immune response [228]. However, HIV-1 can evade these mechanisms, survive, and efficiently replicate in macrophages, as described in detail above.

### 4.2. Macrophages and HIV-1 during Late Chronic Phase of Infection

In the later stages of HIV-1 infection, there is a shift of macrophage phenotypes from the M1 pro-inflammatory to M2 anti-inflammatory phenotype due to the recruitment and activation of T cells and their massive secretion of IL-4 and IL-13 (Figure 5) [223]. Moreover, unlike HIV-infected CD4+ T cells, which die within a few days of infection, infected macrophages resistant to the cytopathic effects of HIV-1 replication are widely distributed in the tissues, and have a longer life span than most cells of the myeloid lineage, ranging from months to years [229,230], resulting in continuous viral replication and production [231].

Additionally, it has been shown that killing of HIV-1-infected macrophages by CD8+ cytotoxic T lymphocytes (CTLs) is impaired compared to the killing of infected CD4+ T cells, and used distinct mechanisms [232]. The rapid killing of CD4+ T cells is caspase independent and does not require granzyme B. In contrast, the killing of macrophages depends on both caspase-3 and granzyme B. The inefficient CTL-mediated killing of macrophages drives prolonged formation of synapses between effector and target cells, accompanied by the higher production and secretion of IFN-γ (a major macrophage-activating cytokine) from CTLs of HIV-infected patients. In turn, interactions between CTLs and macrophages result in high expression levels of pro-inflammatory cytokines from macrophages of HIV-infected donors that favor the recruitment of other immune cells such as monocytes and T cells [232]. Moreover, it has been shown that HIV-1 infection of macrophages significantly stabilizes their interactions with CD4+ T cells compared to uninfected macrophages for more efficient virus cell-to-cell spreading and *cis*-infection of target CD4+ T cells [216,217,232]. Together, the combination of these factors supports the progression of immune dysregulation, creating conditions conducive to opportunistic infections, autoimmune diseases, and malignancies (Figure 5) [232,233].

Chronic inflammatory processes are involved both in tissue destruction and repair [234]. This dynamic results in a positive feedback loop of immune responses and damage. Chronic inflammation is a major contributor to the pathophysiology of neoplastic, neurodegenerative, autoimmune, and cardiovascular diseases. Similarly, systemic chronic immune activation and inflammation are featured as an in vivo hallmark of HIV-1 infection. This could lead to an increased risk of HIV-associated non-AIDS complications and the dysfunction of T cells, despite long-term viral suppression by cART and restoration of CD4+ T cell levels [235,236,237,238,239].

Immune activation and inflammation persist in the majority of treated HIV-infected individuals and are associated with a high risk of mortality and morbidity. While many factors contribute to this aberrant immune activation in vivo, the persistent infection of myeloid cells, most likely tissue-resident macrophages, is postulated to contribute to the chronic immune activation and inflammation. This was notably evidenced in cART-treated HIV-1-infected humanized mice [80,82], although the molecular mechanisms of how HIV-1 replication activates macrophages remain poorly understood. Macrophage activation and secretion of proinflammatory cytokines and chemokines, such as IP-10 (also known as CXCL10) and type I IFNs, caused by HIV-1 infection may have a significant impact on pathogenesis.

IP-10 is one of the most abundant chemokines produced by HIV-1-infected macrophages [235], and IP-10 levels are highly associated with HIV-1 disease progression through suppressing immune cell functions and facilitating HIV-1 replication and dissemination [240]. IP-10 was also shown to induce HIV-1 latency in resting CD4^+^ T cells. The exposure of latently infected CD4^+^ T cells to immune activation stimuli can also result in spontaneous reactivation of viral transcription, which may lead to localized replication of HIV-1 in tissues and contribute to blips of viremia. Type I IFNs secreted by activated macrophages may be involved in the activation of viral transcription in CD4+ T cells [241], suggesting that HIV-infected macrophages may contribute to viral transcription in latently infected CD4+ T cells. Moreover, the persistent expression of HIV-1 intron-containing RNA in macrophages promotes an immune exhaustion phenotype in co-cultured CD4+ and CD8+ T cells in an IFN-I-dependent manner [235].

In chronic HIV-1 infection, the persistent exposure to IFN-I has been indicated to be the driver of T cell activation, resulting in the loss of immunological control of HIV-1-infected cells, and thus contributing to HIV-1 persistence and reactivation [242,243]. Persistent IFN-I signaling is the major contributor to this persistent inflammation and the subsequent immune activation are associated with worsened disease progression in chronic infections (Figure 5) [244]. It has been proposed that HIV-1 infection of macrophages triggers the production of inflammatory cytokines [245,246,247,248] and ISG expression [245,249,250,251] without measurable IFN-I production, at least by classical ELISA. However, recent studies have suggested, using ultra-sensitive digital ELISA, that early elevated IFN-α levels in patients’ serum could serve as a key marker of HIV-1 pathogenesis. In this study, the authors reported that elite controller patients who did not progress to AIDS exhibited lower levels of serum IFN-α than progressor patients [252]. In monkey models of SIV infection, infected animals that do not progress to AIDS despite ongoing high virus replication have lower IFN-I signaling signatures. In contrast, AIDS-progressing non-natural host monkey species, such as macaques, exhibit high levels of sustained IFN-I signaling, independent of the viral load [253,254,255]. These observations suggest that, in addition to promoting antiviral immunity, the chronic inflammation mediated by IFN-I leads to progressive immune dysfunction and disease, separate from the effects of virus replication [256,257,258].

Mechanistically, microbial translocation across the intestinal mucosa is the best-established cause of the systemic inflammation observed in people living with HIV-1 [259,260,261]. Indeed, myeloid cell-derived biomarkers of microbial translocation, such as IL-6, soluble CD14 (sCD14), or sCD163, are elevated in cART-treated individuals compared to age-matched controls, and are strongly associated with premature mortality of HIV-infected patients [262]. Chronic macrophage activation and potential dysfunction are not immediate consequences of HIV-1 infection. Whereas plasma levels of sCD163 released from activated monocytes/macrophages induced by microbial ligands is normalized in patients treated early with effective cART after HIV-1 primo-infection (≤1 year in duration), sCD163 levels decreased in parallel with plasma viral loads but did not return to HIV-seronegative levels in treated individuals with a chronic HIV-1 infection (>1 year in duration), highlighting a persistent residual activation of monocytes/macrophages [263]. These persistent elevated plasma sCD163 levels in individuals with a chronic infection may be a marker of HIV-1 disease activity in long-lived cells such as macrophages [263,264]. This persistent activated state appears to have a feedback effect on intestinal macrophages, as they are unable to engulf microbial debris in the lamina propria and thus are unable to stop this inflammatory cycle [6,265].

The immune dysregulation observed in chronic HIV-1 infection is therefore intricately linked to the dynamic responses elicited by infected macrophages. These cells not only contribute to the chronic inflammatory environment through the release of pro-inflammatory cytokines and chemokines, but they also exhibit compromised functional capabilities. The impaired phagocytic activity and altered antigen presentation by HIV-infected macrophages contribute significantly to the overall immune dysfunction characteristics of advanced stages of the disease.

## 5. Concluding Remarks

Over the past decade, myeloid cells, including tissue macrophages and dendritic cells, have emerged as important target cells of HIV-1, and are probably involved in all steps of the infection’s pathogenesis. Infected macrophages, together with CD4+ T cells and dendritic cells that are also found in genital and rectal mucosae, could be among the first cells targeted by HIV-1 and in contact with transmitted viruses during sexual transmission. Furthermore, infected macrophages then participate in early virus dissemination in lymphoid and nonlymphoid tissues, often as multinucleated giant cells, where they can definitively play a major role in the early establishment of long-lived virus reservoirs in numerous tissues even in people living with HIV on effective highly active cART, as confirmed in experimentally SIV-infected macaques and HIV-1-infected humanized mice. Moreover, growing evidence suggests that they also certainly participate, as key regulators of innate immunity and inflammation, directly or indirectly in the chronic inflammation observed in people living with HIV during the different stages of HIV-1 pathogenesis.

As shown in this review, macrophages are paradoxically more difficult to infect in vitro with cell-free virus particles than activated primary CD4 T cells. This apparent block in virus replication is partly related to host cell restriction factors, such as SAMHD1, which are highly expressed in myeloid cells and inhibit the post-entry steps of the viral life cycle; however, the first block to macrophage infection in vitro is related to virus entry and the specific cellular tropism of the HIV-1 variants found in infected individuals. Notably, several recent studies showed that the different mechanisms of virus cell-to-cell transfer from infected CD4 T cells described above may represent the dominant modes of virus dissemination in vivo for the productive infection of myeloid cells, including resident tissue macrophages. Importantly, these modes of cell-to-cell virus dissemination may enable the virus to escape neutralization by anti-envelope neutralizing antibodies, reduce their sensitivity to antiretroviral drugs, and finally bypass or overcome the restriction by host cell factors specifically acting at the different steps of the virus life cycle when macrophages are infected by cell-free virus particles.

While in vivo access to biological tissue material containing infected macrophages is quite challenging in patients living with HIV, it is important to further characterize and decipher, first in vitro, the specific cellular and molecular mechanisms used by HIV-1 to circumvent the host cell restriction factors limiting the entry and post-entry steps of the viral life cycle. This research aligns with the early presence and detection of HIV-infected macrophages and multinucleated myeloid cells in a wide range of tissues in infected patients. More importantly, there is an urgent need for further in vivo investigations on experimental animal models, such as SIV-infected monkeys or HIV-1-infected humanized mice, for a better understanding and characterization of the direct and indirect roles played by macrophages in chronic inflammation and HIV-1 pathogenesis. These combined in vitro and in vivo investigations, using more recent experimental techniques and tools and reagents that are more specific for the myeloid lineage, should provide essential information about the specific role of myeloid target cells, particularly macrophages, in viral transmission, early viral spreading in different lymphoid and non-lymphoid tissues, and finally in the establishment and persistence of infected macrophages as tissue viral reservoirs, the major obstacle to virus eradication in people living with HIV.

Gaining a thorough understanding of the intricate interactions between macrophages and HIV-1 infection stands as a critical foundation for the development of therapeutic approaches aimed at effectively targeting viral reservoirs and alleviating the immune dysregulation inherent to chronic HIV-1 infection. Ongoing research in this area should provide a better understanding of the complex dynamics that characterize the interactions between HIV-1 and macrophages. The current research initiatives in this area are indispensable for unraveling the ever-evolving complexities of HIV–macrophage interactions. This exploration will delve into the molecular intricacies governing viral entry, replication, and evasion within macrophages.
